# Unraveling Myelin Plasticity

**DOI:** 10.3389/fncel.2020.00156

**Published:** 2020-06-11

**Authors:** Giulia Bonetto, Yasmine Kamen, Kimberley Anne Evans, Ragnhildur Thóra Káradóttir

**Affiliations:** ^1^Wellcome – Medical Research Council Cambridge Stem Cell Institute and Department of Veterinary Medicine, University of Cambridge, Cambridge, United Kingdom; ^2^Department of Physiology, Biomedical Centre, Faculty of Medicine, University of Iceland, Reykjavik, Iceland

**Keywords:** myelin plasticity, oligodendrocyte, oligodendrocyte precursor cell, myelin, glutamate

## Abstract

Plasticity in the central nervous system (CNS) allows for responses to changing environmental signals. While the majority of studies on brain plasticity focus on neuronal synapses, myelin plasticity has now begun to emerge as a potential modulator of neuronal networks. Oligodendrocytes (OLs) produce myelin, which provides fast signal transmission, allows for synchronization of neuronal inputs, and helps to maintain neuronal function. Thus, myelination is also thought to be involved in learning. OLs differentiate from oligodendrocyte precursor cells (OPCs), which are distributed throughout the adult brain, and myelination continues into late adulthood. This process is orchestrated by numerous cellular and molecular signals, such as axonal diameter, growth factors, extracellular signaling molecules, and neuronal activity. However, the relative importance of, and cooperation between, these signaling pathways is currently unknown. In this review, we focus on the current knowledge about myelin plasticity in the CNS. We discuss new insights into the link between this type of plasticity, learning and behavior, as well as mechanistic aspects of myelin formation that may underlie myelin plasticity, highlighting OPC diversity in the CNS.

## Introduction

**FIGURE 1:**
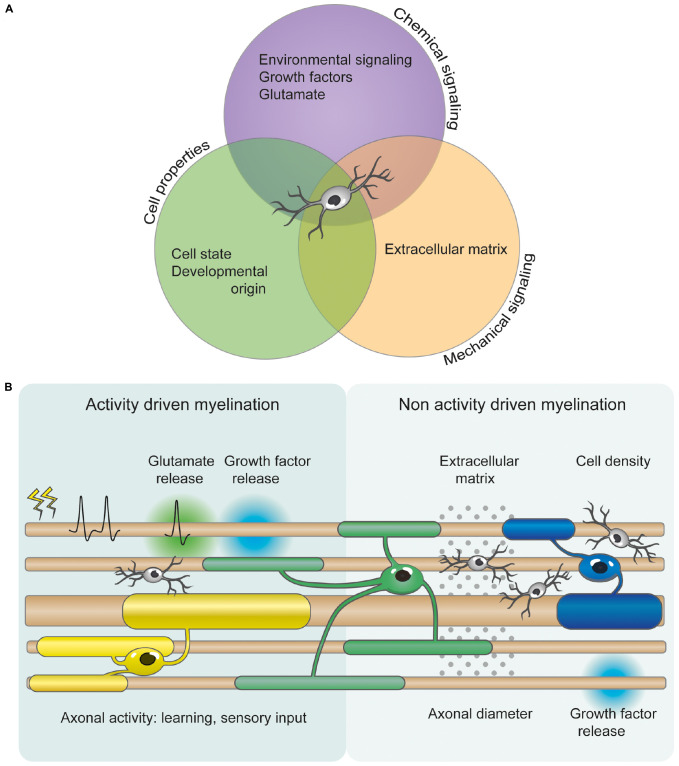
OPC heterogeneity and axonal factors allow for differential myelination and myelin plasticity. **(A)** OPC proliferation, differentiation and myelination are orchestrated by numerous mechanical, cellular, and chemical signals. These include axonal diameter, growth factors, extracellular signaling molecules, extracellular matrix composition, cellular intrinsic deposition, neurotransmitters (such as glutamate), and neuronal activity. However, the relative importance of and cooperation between these signaling pathways is currently unclear. **(B)** Several studies indicate that myelination can be modified by activity- and experience-driven mechanisms. Glutamate and growth factor release from electrically active neurons can regulate OPC proliferation, differentiation and myelination. Additionally, motor and possibly cognitive learning, and sensory experience also influence myelination changes. However, myelination can also occur independently of neuronal activity. Non activity driven myelination could be regulated by the physical and mechanical properties of the extracellular environment, such as cellular density and extracellular matrix. OPCs are depicted in light gray. OLs are represented in different colors to illustrate the differential myelination.

In the central nervous system (CNS), myelin is produced by oligodendrocytes (OLs) that differentiate from oligodendrocyte precursor cells (OPCs) (Fields, 2014). OPCs are distributed throughout the adult brain and represent the main self-renewing population of cells in the CNS (Dawson et al., 2003). Myelination has generally been studied in a developmental context and is often described as a process that terminates after juvenile development. However, recent work shows that myelination continues into late adulthood, with adult OPCs providing a continuous supply of new myelinating OLs (Rivers et al., 2008; Hughes et al., 2013, 2018; Young et al., 2013; Hill et al., 2018). This suggests that protracted myelination may allow for fine-tuning of neural circuits throughout life. While studies of brain plasticity mostly focus on neuronal synapses, myelin plasticity, defined as the myelination of previously unmyelinated axons or changes in the structure of already-myelinated axons (e.g., ion channel surface expression, changes in internode number and length, myelin thickness or geometry of the nodal area), is now also thought to modulate neural networks (Sampaio-Baptista et al., 2013; Gibson et al., 2014; McKenzie et al., 2014; Pajevic et al., 2014). Indeed, changes in myelin sheath stability, length and thickness can alter conduction velocity, and therefore modulate input synchronization (Waxman, 1997; Fields, 2015). While myelin plasticity is a novel field of study, and the mechanisms underlying it are poorly understood, it is likely that several processes governing developmental myelination are applicable in the context of plasticity (Figure 1). In particular, developmental myelination is thought to occur either in a neuronal activity-independent or -dependent mode (Lundgaard et al., 2013). Here, we will briefly review both modes of myelination, along with the role of motor, cognitive and sensory learning, and OPC diversity, in the context of myelin plasticity.

## The Differential Path of Myelination

### Activity-Independent Myelination

Developing OPCs are proliferative, self-renewing cells that possess the capability to differentiate into myelinating OLs (Rosenberg et al., 2008). Notwithstanding, several external cues regulate this differentiation capacity. Neuronal activity, through neurotransmitter signaling, is a regulatory signal for OPC proliferation and differentiation. However, OPCs can differentiate into OLs that wrap inert fibers with compact myelin, and have internodes of expected lengths, clearly indicating that the initiation of OL differentiation and some forms of myelination does not require neuronal activity (Rosenberg et al., 2008; Lee et al., 2012) (Table 1). Nevertheless, the presence of the axon, or an axon-like structure, remains a strong inductive signal for differentiation, suggesting that the biophysical characteristics of the axon, such as the shape and the caliber, regulate OPC differentiation. Axon caliber has been shown to influence myelin thickness (Voyvodic, 1989) and the internodal distribution (Trapp and Kidd, 2000). Similarly, increasing axonal diameter by knocking out *Pten* in axons induces myelination of normally unmyelinated parallel fibers (Goebbels et al., 2017), and retinal ganglion axons following enucleation (i.e., the surgical removal of one eye) (Mayoral et al., 2018). Eye enucleation in a non-degenerative mouse model reduces axonal diameter and myelination, supporting the notion that axon caliber is a main regulatory factor of myelination. Although there is a correlation between axon diameter and myelination, it is important to note that knocking out *Pten* alters growth factor signaling (Goebbels et al., 2017), and that enucleation alters spontaneous firing in the control eye (Failor et al., 2018), raising the possibility that diameter alone is not the only mechanism regulating myelination (Friede, 1972; Lee et al., 2012), or that different axons are myelinated by different mechanisms (Koudelka et al., 2016). Additionally, diameter alone does not explain how the same axon can be differentially myelinated along its length, nor how axons of the same diameter can be either myelinated or remain unmyelinated (Tomassy et al., 2014). Using a neuron-free *in vitro* system in which OPCs from either the spinal cord or cortex differentiate into OLs that ensheath inert fibers, it has been found that spinal cord OLs produced longer sheaths along the microfibers than cortical OLs, consistent with their length in the CNS (Bechler et al., 2015). These data suggest that sheath length may be intrinsic, region-specific, and programmed before differentiation. Intriguingly, Schwann cells, the myelinating glia of the peripheral nervous system (PNS), were not able to myelinate the microfibers, implying that the capacity to myelinate without axonal cues is exclusive to the CNS. However, Schwann cells usually myelinate larger diameter axons than OLs, thus another interpretation could be that the size of the fibers used in this study was insufficient to initiate Schwann cell myelination. This region-specific property points to local cues which regulate OL lineage progression and OL properties.

**TABLE 1:** Summary of current literature on activity-independent and activity-dependent myelination in the CNS.

Myelination modes	Activity-independent	Biophysical properties of the axon	Friede, 1972 Voyvodic, 1989 Fukui et al., 1991 Colello et al., 1995 Shrager and Novakovic, 1995 Trapp and Kidd, 2000 (review) Lee et al., 2012 Tomassy et al., 2014 (provides evidence that biophysical constraints alone cannot explain differential myelination) Goebbels et al., 2017 Mayoral et al., 2018
		Microenvironmental characteristics	Raff et al., 1988 Richardson et al., 1988 Maniotis et al., 1997 Rosenberg et al., 2008 Hernandez et al., 2016 Segel et al., 2019
		Cell properties	Hill et al., 2013 Bechler et al., 2015
	Activity-dependent	Neuronal regulation of OPC proliferation and differentiation, and myelination	Gyllensten and Malmfors, 1963 Tauber et al., 1980 Barres and Raff, 1993 Demerens et al., 1996 Stevens et al., 1998 Liu et al., 2012, 2016 Makinodan et al., 2012 Mangin et al., 2012 Gibson et al., 2014 Hill et al., 2014 Mensch et al., 2015 Gautier et al., 2015 Etxeberria et al., 2016 Koudelka et al., 2016 Mitew et al., 2018 Ortiz et al., 2019
		Glutamate signaling	Gallo et al., 1996 Yuan et al., 1998 Bergles et al., 2000 Karadottir et al., 2005, 2008 Micu et al., 2006 Kukley et al., 2007 Ziskin et al., 2007 De Biase et al., 2011 Wake et al., 2011 Cavaliere et al., 2012 Guo et al., 2012 Li et al., 2013 Lundgaard et al., 2013 Fannon et al., 2015 Gautier et al., 2015 Saab et al., 2016 Spitzer et al., 2016 (review) Kougioumtzidou et al., 2017 Spitzer et al., 2019

*This table summarises the reviewed literature, showing selected papers which provide support (if not indicated otherwise) for the mechanisms shown.*

The physical properties of the microenvironment, such as proximity to an axon or cellular density, may induce differentiation by altering OPC size or shape, thus generating structural rearrangement within the cells (Ingber, 1997; McBeath et al., 2004). This rearrangement could allow for interactions between different effectors in a signaling pathway, and thus, promote differentiation (Boudreau and Jones, 1999). Another potential explanation is that changes in cell shape might directly modify the nuclear size or structure, inducing the transcriptional activity necessary for OPC differentiation (Maniotis et al., 1997). This possibility is supported by work showing that mechanically deforming OPCs or plating them in the presence of neurons, beads, or at high density, promotes differentiation by altering the chromatin structure (Hernandez et al., 2016).

A recent study by the Chalut group further supports the idea that the mechanical environment modulates OPC function. By mimicking the stiffness of young brains using scaffolds in culture, they demonstrated that OPCs isolated from aged rats and cultured in these softer conditions became molecularly and functionally similar to neonatal OPCs. Disrupting mechanical signaling in these aged OPCs increased their proliferation and differentiation rate, indicating that increasing brain stiffening with age downregulates the proliferation and differentiation potential of OPCs (Segel et al., 2019). During development, the maturation of the extracellular matrix (ECM) stabilizes neural networks by limiting changes in synaptic connectivity (Bikbaev et al., 2015). Conversely, removing the ECM promotes synaptic plasticity (Lazarevich et al., 2020). It is possible that ECM maturation also limits differentiation and myelination rates with aging to prevent hypermyelination and stabilize neural networks. However, local changes in the ECM may allow for local differentiation and could therefore be a mechanism underlying myelin plasticity.

Changes in the physical environment can also affect the chemical signaling by altering the extra cellular volume, for instance altering growth factor concentration. This could influence OPC development as platelet-derived growth factor (PDGF) activates the α receptor (PDGFRα) on OPCs and regulates both their proliferation and survival (Raff et al., 1988; Richardson et al., 1988; Barres and Raff, 1993). However, not all OPCs respond equally to PDGF. Although PDGFRα protein expression is similar in both gray and white matter OPCs, cells in the white matter of early postnatal organotypic slice cultures proliferate more in response to PDGF than those in the gray matter (Hill et al., 2013). Consistent with this finding, it has been shown that while all adult OPCs continue to divide, white matter cells divide at a higher rate than gray matter cells (Young et al., 2013). It could be argued that the differential response of gray and white matter OPCs to PDGF stems from the microenvironment (physical properties) rather than a cell intrinsic process. Addressing this question, the Nishiyama group showed, by using small tissue section transplant experiments, that regional identity, and not environment, determined the proliferative response to PDGF (Hill et al., 2013). On the other hand, studies looking into differences due to the developmental origin of OPCs, with a transgenic approach (Psachoulia et al., 2009), or regional identity using cell transplantation (Vigano et al., 2013), have failed to find differences in OPC proliferation. A possible explanation of the difference in results is that using small tissue sections, instead of isolated cells, may have provided sufficient environmental signals of the original region to influence OPCs’ response to PDGF in the transplanted area, and, given a longer period of time for the section to integrate into the host slice, these experiments may have yielded a different result. These studies show that physical properties and environment influence OPC proliferation, differentiation and myelination. Although it is unclear whether these properties can mediate myelin plasticity in response to learning and sensory inputs, their contribution cannot be ruled out. Another mechanism such as neuronal activity, known to influence physical properties (Lazarevich et al., 2020), release of growth factors (Barres and Raff, 1993; Balkowiec and Katz, 2000) and regulate myelination, is perhaps more amenable to plasticity changes, as we shall discuss in the next paragraph.

### Activity-Dependent Myelination (Glutamate Signaling)

Numerous studies have shown that neuronal activity can regulate myelination (Table 1). In addition to growth factors, glutamate release from active neurons is a likely mechanism underlying activity-dependent myelination, as OPCs receive synaptic inputs from neurons and express glutamate receptors (Bergles et al., 2000; Karadottir et al., 2005, 2008; Micu et al., 2006; Kukley et al., 2007; Ziskin et al., 2007; Lundgaard et al., 2013; Gautier et al., 2015; Spitzer et al., 2019), allowing them to monitor and respond to neuronal activity. However, it is important to note that OPCs display a range of electrophysiological profiles, with ion channel and glutamate receptor densities varying with age and brain region. OPCs acquire voltage-gated potassium channels (K_V_) and sodium channels (Na_V_), α-amino-3-hydroxy-5-methyl-4-isoxazolepropionic acid receptors (AMPARs), kainate receptors (KARs), and N-methyl-D-aspartate receptors (NMDARs) during development, but at different rates, which appear to be regulated by the environment (Spitzer et al., 2019).

Neuronal activity regulates OPC proliferation *in vivo* (Barres and Raff, 1993; Mangin et al., 2012; Gibson et al., 2014; Mitew et al., 2018) (Table 1). Numerous studies show that blocking activity blocks proliferation (Barres and Raff, 1993), while increasing activity promotes proliferation (Gibson et al., 2014; Mitew et al., 2018). Axonal activity also regulates differentiation (Table 1): decreasing activity by whisker removal or raising mice in social isolation impedes differentiation (Liu et al., 2012, 2016; Makinodan et al., 2012; Hill et al., 2014), although ocular deprivation leads to enhanced differentiation, albeit with reduced internode length (Etxeberria et al., 2016). However, it is important to note that ocular deprivation did not block neuronal firing, but rather altered it (Etxeberria et al., 2016), suggesting that changes in activity modulate differentiation. Similarly, enhancing activity with optogenetics, chemogenetics, receptor agonists/antagonists, or physiological manipulations, promotes differentiation (Gibson et al., 2014; Mitew et al., 2018), or enhances myelination (Tauber et al., 1980; Demerens et al., 1996; Mensch et al., 2015; Mitew et al., 2018). However, other studies using similar approaches have failed to show an effect of neuronal activity on developmental myelination (Fukui et al., 1991; Colello et al., 1995; Shrager and Novakovic, 1995). Nonetheless, blocking neuronal activity decreases myelination and prevents myelin repair after demyelination (Gyllensten and Malmfors, 1963; Demerens et al., 1996; Stevens et al., 1998; Gautier et al., 2015; Mensch et al., 2015; Mitew et al., 2018) while enhancing activity improves remyelination (Ortiz et al., 2019).

Neuronal activity not only promotes myelination through glutamate signaling, but also induces a switch to the activity-dependent mode of myelination. Indeed, activity-dependent release of neuregulin (NRG) or brain-derived neurotrophic factor (BDNF) enhances NMDAR functional expression in OPCs and switches myelination to an activity-dependent mode in neuron-OPC co-cultures (Lundgaard et al., 2013). Activity-dependent myelination occurs faster than activity-independent myelination, and crucially, blocking neuronal activity, NMDARs, or AMPARs once the activity-dependent mode is activated by NRG or BDNF significantly reduces myelination (Lundgaard et al., 2013). However, blocking activity, NMDARs or AMPARs does not affect myelination in the absence of NRG or BDNF (Lundgaard et al., 2013). In mice, blocking activity-dependent BDNF release or deleting the BDNF receptor TrkB in OPCs blocks activity-dependent myelination (Geraghty et al., 2019). Nevertheless, NRG/ErbB (the NRG receptor) signaling plays a complicated role in the CNS (Lyons et al., 2005; Brinkmann et al., 2008). Despite evidence indicating a function in OL differentiation, myelination, and survival *in vitro* (Flores et al., 2000; Kim et al., 2003; Taveggia et al., 2008), knocking out ErbB3 and ErbB4 in OL lineage cells does not prevent myelination (Brinkmann et al., 2008), although it is unclear whether there was a delay in myelination, it does seem to prevent experience-dependent myelination (Makinodan et al., 2012). The controversial actions of NRG/ErbB signaling can be explained by the ability of NRG to switch OPCs from activity-independent myelination to the activity-dependent mode through the increase of NMDAR-mediated currents in OL lineage cells (Lundgaard et al., 2013). It is likely that in the absence of ErbB3 and ErbB4, NRG could not enhance NMDAR expression in OPCs, and therefore, could not induce the switch to activity-dependent myelination. Therefore, a delay in myelination, but no myelination defect, would be expected, although this was not tested. Moreover, the release of NRG itself is activity-dependent (Ozaki et al., 2004). The resulting interaction of NRG with glutamate signaling increases myelination on active neurons, providing a mechanism by which activity plays a role in myelination and myelin plasticity (Spitzer et al., 2016).

The importance of glutamate signaling in activity-dependent myelination is revealed by studies of myelin repair following demyelinating lesions. Blocking neuronal activity, vesicular release, AMPARs or NMDARs at the lesion site impedes myelin regeneration after toxin-induced demyelination (Lundgaard et al., 2013; Gautier et al., 2015). However, the exact role of glutamate signaling *in vivo* remains elusive. *In vitro* studies report that AMPA/KAR activation reduces both proliferation and differentiation (Gallo et al., 1996; Yuan et al., 1998; Fannon et al., 2015), while activating NMDARs promotes myelination (Wake et al., 2011; Cavaliere et al., 2012; Li et al., 2013; Lundgaard et al., 2013). Nevertheless, the importance of glutamate signaling through AMPARs and NMDARs for myelination *in vivo* is disputed due to the mild deficits observed in the respective knockouts (De Biase et al., 2011; Guo et al., 2012; Saab et al., 2016; Kougioumtzidou et al., 2017). However, both receptors may have been knocked out in OPCs prior to the activation of AMPA/KARs or NMDARs (Spitzer et al., 2019). Even if neurons released NRG or BDNF onto OPCs, myelination would not have switched to the activity-dependent mode, as OPCs did not have NMDARs or AMPARs, and therefore, NRG could not enhance NMDAR expression. If this were the case, a delay in myelination would be expected, due to compensation by the slower activity-independent mode of myelination. While this was not tested in all of the studies above, two groups found that both the AMPAR and NMDAR knockouts lead to a delay in myelination (Saab et al., 2016; Kougioumtzidou et al., 2017). As activity-independent myelination occurs slower than activity-dependent myelination, these studies indicate that it is possible that compensation may have occurred by defaulting to the slower activity-independent myelination mode. Whereas, altering AMPAR receptors, postnatally, during the peak of the myelination period increased OPC proliferation while reducing their differentiation (Chen et al., 2018), suggesting that modifying receptor properties at specific timepoints can alter OPC dynamics. These studies indicate that glutamate signaling through AMPA/KARs and NMDARs depends on a complex interplay of factors, such as the receptor subtype and density, the frequency, the amount, and probability of glutamate release from active neurons. Nonetheless, glutamate signaling remains an integral mechanism of activity-dependent myelination in the context of both normal developmental myelination and myelin plasticity.

## OPC Heterogeneity

Myelin plasticity includes both *de novo* myelination and structural changes to existing myelin. *De novo* myelination is thought to occur through the differentiation of adult OPCs, which receive cues – presumably axon-derived – following motor, sensory or social experience. Thus, to study myelin plasticity, we must investigate how OPCs integrate these cues. This is made more complex by several groups reporting that OPCs are a heterogeneous population, with differences in their proliferation and differentiation potentials with age or between brain regions (Rivers et al., 2008; Vigano et al., 2013; Young et al., 2013; Moshrefi-Ravasdjani et al., 2017; Spitzer et al., 2019). In addition, bulk-RNA sequencing shows that OPCs exhibit age- related changes in transcriptome (Marques et al., 2018; Spitzer et al., 2019), and single-cell experiments suggest that proliferation and differentiation gene expression is altered with age (Marques et al., 2016, 2018). OPCs also display differential responses to growth factors and cytokines (Mason and Goldman, 2002; Lin et al., 2009; Hill et al., 2013; Lentferink et al., 2018). Furthermore, in zebrafish, two populations of OPCs were identified in the spinal cord: a population that mostly proliferates in response to activity, but does not differentiate, and a second population arising from the first one, which differentiates into myelinating OLs (Marisca et al., 2020). These differences must be considered, especially when attempting to study myelin plasticity through the lens of developmental myelination (Table 2).

**TABLE 2:** Reviewed literature on OPC heterogeneity.

OPC heterogeneity	Differences in transcriptomics	Marques et al., 2016, 2018 Spitzer et al., 2019 Marisca et al., 2020
	Differential response to growth factors and cytokines	Mason and Goldman, 2002 Lin et al., 2009 Hill et al., 2013 Lentferink et al., 2018
	Region and age-dependent changes in physiological properties	Chittajallu et al., 2004 Karram et al., 2008 De Biase et al., 2010 Clarke et al., 2012 Moshrefi-Ravasdjani et al., 2017 Spitzer et al., 2019 Marisca et al., 2020
	Diverse proliferation and differentiation potential	Rivers et al., 2008 Vigano et al., 2013 Young et al., 2013 Marques et al., 2016 Moshrefi-Ravasdjani et al., 2017 Spitzer et al., 2019 Marisca et al., 2020

*This table summarises the reviewed literature showing selected papers describing OPC heterogeneity. The references in blue suggest that OPCs are physiologically homogeneous.*

Given the role of neuronal activity, via glutamate signaling, in regulating OPC proliferation, differentiation, and myelination, and its potential role in myelin plasticity regulation, it is important to understand if all OPCs display the same physiological properties. While some reports indicate that OPCs from the hippocampus and corpus callosum are homogeneous (De Biase et al., 2010; Clarke et al., 2012), differences in ion channels between gray and white matter OPCs have been described (Chittajallu et al., 2004; Spitzer et al., 2019). Furthermore, age-dependent changes in ion channels have also been described (Karram et al., 2008; Moshrefi-Ravasdjani et al., 2017; Spitzer et al., 2019) (Table 2). An in-depth study of mouse OPC membrane properties in different brain regions between embryonic day 13 and postnatal day 330 indicates that the density of Na_V_, K_V_, AMPA/KARs and NMDARs differs. Specifically, at embryonic day 13, when they first appear in the brain, OPCs have no ion channels or glutamate receptors, and acquire them with age at different rates, and differentially between brain regions (Spitzer et al., 2019). Functional expression of ion channels and glutamate receptors can be linked to the proliferation and differentiation potential of OPCs (Spitzer et al., 2019).

These data led to the identification of several OPC states. First, embryonic-like “naïve” OPCs, lacking ion channels and glutamate receptors, which cannot sense neuronal activity. Second, “highly proliferative” OPCs, with K_V_, AMPA/KARs, and a high density of Na_V_. Third, OPCs that are “primed” for differentiation, with K_V_, AMPA/KARs, a high Na_V_ density, and a high density of NMDARs, indicative of a high sensitivity to neuronal activity. Lastly, “quiescent” OPCs, who have lost NMDARs, and have acquired a high density of AMPA/KARs (Spitzer et al., 2019). Importantly, at every postnatal time point and brain region tested, a range of electrophysiological profiles of OPCs can be detected, although in differing proportions, suggesting that this functional diversity may represent cell states rather than heterogeneity.

Understanding OPC states is crucial for our understanding of both activity-dependent and activity-independent myelination. For instance, most embryonic OPCs are naïve, yet proliferate, and, in the spinal cord, have begun to differentiate, perhaps indicating that early developmental myelination may proceed in an activity-independent mode, presumably to ensure that critical processes like breathing are functional by birth (Foran and Peterson, 1992; Spitzer et al., 2019). In addition, the majority of OPCs are in the primed state during the first three postnatal months, at the time where differentiation and myelination are proceeding at the highest rate, and NMDAR expression is highest, suggesting that at this time, myelination is activity-dependent (Spitzer et al., 2019). Most studies on motor or sensory myelination and myelin plasticity have been performed at this time. It is therefore not surprising that activity-dependent myelination is thought to underlie myelin plasticity.

This poses the problem of what happens in mature brains, once most OPCs have become quiescent. Does myelination stop, and does myelin plasticity remain possible? Is plasticity limited to a critical window, defined by OPC ion channel expression? The majority of OPCs were described as quiescent by nine months, yet OPC differentiation and myelination have been reported to continue in the mouse cortex until 2 years of age (Young et al., 2013; Hill et al., 2018; Hughes et al., 2018; Spitzer et al., 2019). In addition, a study examining plasticity in adult mice showed that sensory enrichment increased the formation of new myelin in the somatosensory cortex of 10–14 month old mice (Hughes et al., 2018). The signaling mechanism driving this plasticity was not investigated, but sensory enrichment alters neuronal activity, which may in turn lead to the release of NRG or BDNF from neurons, promoting NMDAR functional expression in OPCs, and a shift to the primed state (Lundgaard et al., 2013; Spitzer et al., 2019). Indeed, glutamate receptors in OPCs can be regulated by growth factors (Gallo et al., 1994; Lundgaard et al., 2013). Thus, growth factors may regulate state transitions, and allow for activity-dependent myelination following major sensory events.

OPC states may also influence the different myelination strategies employed by different brain regions. For instance, in the rodent optic nerve, myelin tends to be remodeled (with changes on already myelinated axons), while in the corpus callosum, the tendency is more toward *de novo* myelination of unmyelinated axons (Young et al., 2013). It is therefore critical that we understand both OPC states and their regulators to better understand myelination, and myelin plasticity.

## Regulation of Myelination by Motor Learning, Social Behavior and Sensory Experience

Recent evidence suggests that myelination may be dynamically regulated by learning and experience, and may therefore play a role in learning (Table 3). Structural changes in human white matter occur with learning new tasks, such as playing the piano (Bengtsson et al., 2005) or learning how to juggle (Scholz et al., 2009), though whether these changes indicate myelin remodeling remains unclear (Zatorre et al., 2012; Walhovd et al., 2014). Nevertheless, experiments combining diffusion MRI fractional anisotropy (as in human studies) and immunohistochemistry have shown that motor learning in adult mice leads to white matter structural changes which correlate with an increased myelin density (Sampaio-Baptista et al., 2013). Furthermore, the Richardson group showed that motor learning increases OPC differentiation into myelinating OLs in the motor cortex and corpus callosum, and that motor learning is in fact dependent on this (McKenzie et al., 2014; Xiao et al., 2016).

**TABLE 3:** Summary of reviewed literature on learning and experience.

Learning and experience	Motor learning	Bengtsson et al., 2005 Scholz et al., 2009 Sampaio-Baptista et al., 2013 McKenzie et al., 2014 Xiao et al., 2016
	Cognitive functions	Carreiras et al., 2009 Schlegel et al., 2012 Geraghty et al., 2019
	Social behavior	Liu et al., 2012 Makinodan et al., 2012
	Sensory experience	Mangin et al., 2012 Hill et al., 2014 Hughes et al., 2018

*Table recapitulating the most relevant studies linking myelin modifications with learning, social behavior and sensory experience. Human studies are indicated in red, while rodent studies are indicated in black.*

A current outstanding question in the field is whether myelination is initiated only during sensory-motor learning or in all types of learning. In human studies, changes in white matter have been observed following reading (Carreiras et al., 2009) or learning a second language (Schlegel et al., 2012), suggesting that modifications in myelin may also occur following cognitive learning. In addition to various reports demonstrating that sensory experience or neuronal activity modulate myelination in the somatosensory system (Hughes et al., 2018; Mitew et al., 2018), a recent publication suggests that myelin plasticity is important for normal cognitive function. Activity-regulated myelination fails in a model of chemo-therapy-related cognitive impairment (CRCI), a syndrome characterized by deficits in attention and memory (Koppelmans et al., 2012), and this is linked to a reduced BDNF-TrkB signaling in OPCs, as demonstrated by the deficits in cognitive behavioral performance following the OPC-specific TrkB receptor loss (Geraghty et al., 2019).

It seems that changes in myelination as a response to the environment have important long-term behavioral and cognitive consequences. Indeed, rearing juvenile mice in social isolation alters myelin in the medial prefrontal cortex (mPFC) (Liu et al., 2012; Makinodan et al., 2012). In one of these studies, NRG was shown to be decreased following social isolation, and the modifications in myelin were phenocopied by an OL ErbB3 receptor knockout (Makinodan et al., 2012). Together, these data indicate that social experience, presumably via neuronal activity, regulates myelination and that this is important for normal cognitive function.

Sensory experience also influences myelin plasticity. Whisker deprivation, by unilateral removal, leads to a decrease in the number of mature OLs, but an increase in OPC density and proliferation (Mangin et al., 2012; Hill et al., 2014). However, whisker deprivation also increases apoptosis of proliferating OPCs (Hill et al., 2014). Thus, these data suggest that whisker deprivation leads to a decrease in mature OL numbers, which may in turn lead to over proliferation of OPCs, although the increase in apoptosis may be a mechanism to maintain homeostasis (Hill et al., 2014).

The surprisingly rapid dynamics of OL production in response to motor learning (within 2 h) (Xiao et al., 2016) and myelin basic protein (MBP) translation in response to neuronal activity (within minutes to hours) (Wake et al., 2011) occur on a timescale that is similar to that of dendritic spine changes underlying synaptic plasticity (Xu et al., 2009). Like synaptic plasticity, myelin plasticity following motor or cognitive learning and sensory experience is thought to be regulated by activity-dependent myelination, as motor, cognitive and sensory events lead to changes in neuronal activity (although the contribution of activity-independent myelination cannot be excluded) (Figure 1). These data suggest that myelin plasticity and synaptic plasticity may be complementary mechanisms underlying learning and memory.

## Outlook and Future Perspective

Until recently, myelination was considered a static process, and studies examining circuit function and plasticity mostly focused on synaptic plasticity. However, a number of studies described above demonstrate that myelination is far from static, and does not only change in response to injury, but also as a result of motor, sensory and cognitive events. Although some progress has been recently made, our knowledge of myelin plasticity and the mechanisms underlying it remains restricted by regional differences, OPC diversity, a lack of understanding of the neuronal dynamics required to regulate OL lineage progression, and a limited comprehension of how OL lineage cells integrate the various activity-independent and activity-dependent signals (Figure 1).

The study of myelin plasticity requires morphological analyses, both at the sub microscopic scale and the macroscopic scale, but also a combination of behavioral, electrophysiological and molecular analyses. This can only be achieved by combining both *in vitro* and *in vivo* experimental models.

One area that deserves major investigation is examining whether activity-dependent myelination proceeds similarly in different brain regions. From the studies reviewed in this paper, it appears that, akin to synaptic plasticity, neuronal activity-dependent myelin plasticity may be an important mechanism underlying learning and cognition. Activity-dependent release of growth factors and glutamate may be particularly important for this process, and thus, it is crucial to understand these mechanisms of myelination. Moreover, dynamic myelin changes in the hippocampus and mPFC, two regions that are comprised of both gray and white matter, are likely to have long-lasting effects on brain function. In humans, myelination of these regions continues for decades, suggesting that lifelong myelination and myelin plasticity tune neuronal networks and regulate normal brain function.

## Author Contributions

All authors listed have made a substantial, direct and intellectual contribution to the work, and approved it for publication.

## Conflict of Interest

The authors declare that the research was conducted in the absence of any commercial or financial relationships that could be construed as a potential conflict of interest.
